# Metabolic Dysfunction in Parkinson’s Disease: Unraveling the Glucose–Lipid Connection

**DOI:** 10.3390/biomedicines12122841

**Published:** 2024-12-13

**Authors:** Jeswinder Sian-Hulsmann, Peter Riederer, Tanja Maria Michel

**Affiliations:** 1 Department of Human Anatomy and Medical Physiology, University of Nairobi, P.O. Box 30197, Nairobi 00100, Kenya; drjsian@gmail.com; 2 Research Unit of Psychiatry, Department of Psychiatry, Odense, Region of Southern Denmark, University Hospital of Southern Denmark, 5000 Odense, Denmark; peter.riederer@uni-wuerzburg.de

**Keywords:** Parkinson’s disease, substantia nigra, neurodegeneration, glucose metabolism, lipid metabolism, mitochondria, pathogenesis, multifactorial, metabolic dysfunction, protein aggregation

## Abstract

Despite many years of research into the complex neurobiology of Parkinson’s disease, the precise aetiology cannot be pinpointed down to one causative agent but rather a multitude of mechanisms. Current treatment options can alleviate symptomsbut only slightly slow down the progression and not cure the disease and its underlying causes. Factors that play a role in causing the debilitating neurodegenerative psycho-motoric symptoms include genetic alterations, oxidative stress, neuroinflammation, general inflammation, neurotoxins, iron toxicity, environmental influences, and mitochondrial dysfunction. Recent findings suggest that the characteristic abnormal protein aggregation of alpha-synuclein and destruction of substantia nigra neurons might be due to mitochondrial dysfunction related to disturbances in lipid and glucose metabolism along with insulin resistance. The latter mechanism of action might be mediated by insulin receptor substrate docking to proteins that are involved in neuronal survival and signaling related to cell destruction. The increased risk of developing Type 2 Diabetes Mellitus endorses a connection between metabolic dysfunction and neurodegeneration. Here, we explore and highlight the potential role of glycolipid cellular insults in the pathophysiology of the disorder, opening up new promising avenues for the treatment of PD. Thus, antidiabetic drugs may be employed as neuromodulators to hinder the progression of the disorder.

## 1. Introduction

### General Aspects of Parkinson’s Disease

Parkinson’s disease (PD) [1] is a progressive neurodegenerative disorder that exhibits abnormal movement, including resting tremor, bradykinesia, akinesia, hypokinesia and rigidity [2]. These are often coupled with non-motor symptoms such as depression and sleep disturbances and cognitive alterations, like Parkinson’s dementia disorder or Lewy body disorder, which may worsen as the disease progresses [3]. The loss of dopaminergic neurons in the substantia nigra (SN) pars compacta contributes mainly to the loss of voluntary movement observed in the disorder. Another characteristic pathological hallmark is the presence of Lewy bodies (LBs), which comprise misfolded alpha-synuclein aggregates. Many processes have been implicated in the destruction of the nigra cells and the generation of LBs [4].

Despite many years of research and some success at treating the symptoms of PD, the causative factors remain to be elucidated. Moreover, the precise mode(s) in which they interact in the complex labyrinth of events is still not completely understood; hence, treatment strategies to prevent neurodegeneration and stop the cascades implied are limited. However, the molecular pathways leading to the clinical symptoms include oxidative stress, neuroinflammation, dysfunction of protein clearance mechanisms/defective endolysosomal pathway, genetic components, and defective mitochondrial function [5]. However, the complex interactions remain to be elucidated more closely. Moreover, increasing evidence points to disruption of mitochondrial functioning, lipid and especially glucose metabolism in neurodegenerative diseases such as Alzheimer’s dementia and, lately, PD. In the following sections, we will give an overview of the implications and key role of metabolism in the complex molecular underpinnings of PD, which is a stepping stone to opening up new avenues for treatment options and targets.

## 2. Specific Aspects of PD and Metabolism: Mitochondria, Glucose, and Lipid Metabolism

### 2.1. Mitochondrial Dysfunction in PD

Langston and colleagues [6] first implicated mitochondrial dysfunction in PD. They described Parkinsonian like symptoms in illicit users of meperidine analogue 1-methyl-4-phenyl-1,2,5,6-tetrahydropyridine (MPTP). The active agent in MPTP is 1-Methyl-4-phenylpyridinium (MPP+), which is a mitochondrial complex 1 inhibitor in dopaminergic neurons.

Subsequently, a marked reduction in the activity of mitochondrial NADH-ubiquinone reductase (complex 1) was found in the SN from post-mortem brain tissue of PD patients [7,8,9]. This observation indicated mitochondrial dysfunction, which represents a crucial pathological factor since it has been reported repeatedly in sporadic and familial forms of illness [10,11].

The underlying cause(s) of PD might be many, but a strong link is seen for mutations in mitochondrial DNA that may alter the physiological dynamics, the presence of some mutated protein associated with mitochondria, or changes in the morphology of PD that may alter its function [12,13]. Mitochondrial dysfunctions can be linked to mutations that may initiate intense oxidative and inflammatory cellular damage and are associated with conditions such as Diabetes, myopathies, and neuropathies [14,15]. Indeed, in familial PD, several genes have been implicated in underlying mitochondrial dysfunction, including PARK 2, 6, 7, and 8 [16,17,18,19].

Furthermore, the mitochondria are a hub for producing toxic superoxide and other free radicals within the cell, released from oxidative phosphorylation processes [20]. This makes the mitochondria vulnerable to free radical-driven cytotoxic processes such as oxidative stress [21,22]. Additionally, since oxidative stress occurs early in the disorder’s progression, it may contribute significantly to mitochondrial dysfunction. Indeed, the depletion of the antioxidant reduced glutathione (GSH) in the SN in asymptomatic PD or incidental Lewy body disease endorses the occurrence of oxidative stress [23].

Mitochondrial dysfunction can contribute to a number of processes that are catastrophic for the cells, such as producing reactive free radicals leading to oxidative stress, alpha-synuclein aggregation, neuroinflammation [24], and disturbances in glucose and lipid metabolism. Of note is that dysfunction of both glucose and lipid metabolism causes a lack of bioenergetic supply of mitochondria [25,26]. Environmental neurotoxins, such as many pesticides, are so-called “uncouplers” of mitochondrial oxidative phosphorylation, resulting in a decrease in adenosine triphosphate (ATP) production [27]. This destroys not only pests and insects but can also cause neurological damage in humans, particularly over long-term exposure.

Thiamine-dependent processes are primarily of importance in glucose metabolism. Thiamine acts as a coenzyme for transketolase and for the pyruvate dehydrogenase and alpha-ketoglutarate dehydrogenase complexes, which play a fundamental role in intracellular glucose metabolism. Thiamine is involved in the development of the mitochondrial membrane and synaptic membrane function [28]. Alpha-ketoglutarate dehydroxygenase is a thiamine-dependent enzyme of the glycolysis pathway, and its activity is decreased in PD [29]. Increased dopamine release has been detected in experimental studies in rat striatum. In addition to that, thiamine, as well as dopamine itself, has been suggested to be a critical factor in increasing oxidative stress and peroxisomal mechanisms, as well as in gene expression and protein processing [30]. Mitochondrial pyruvate dehydrogenase and 2-oxoglutarate dehydrogenase are of major importance in delivering ATP, a primary source of energy supply for all neuronal tissues.

Additionally, nigral mitochondrial malfunction may exacerbate the production of lactate, a by-product of glycolysis. Subsequently, lactate can evoke neuronal destruction, perhaps via prompting or augmenting microgliosis and inflammatory-mediated cytotoxic processes that encourage misfolding of alpha-synuclein. Lactate may also affect other metabolic pathways, such as lipid metabolism, producing toxic lipid droplets that tend to cause neuronal destruction via lipid peroxidation and reactive oxygen-mediated oxidative stress. Thus, an imbalance in glucose homeostasis can have dire consequences on cellular bioenergetics and may affect the metabolism of lipids. The dysfunction of glucose metabolism may produce cellular energy failure, which may hinder general cellular functions of the dopaminergic neurons, such as neurotransmitter release/metabolism, vesicle transport, protein quality control, and neuron/glial survival. Mitochondrial malfunction may represent an important pathological feature that can disturb glucose metabolism and produce reactive oxygen species that prompt neurotoxic reactions, including oxidative stress and neuroinflammation. Additionally, it can contribute to the accumulation of misfolded protein (alpha-synuclein) aggregates.

This article reviews evidence of metabolic dysfunction, mainly glucose and lipid metabolism, as a potential candidate in the pathophysiology of PD. The evidence strongly supports the involvement of impaired cellular bioenergetics in neurodegeneration. This offers new pharmacological avenues for managing the illness, targeting the impaired glucose/lipid metabolism processes. Therefore, new lines of treatment might be hindering metabolic dysfunction-associated cell death. However, such novel treatments would be an add-on to gold-standard levodopa treatment. Diabetes is an ideal model to provide a better understanding of the consequences of disturbed glucose homeostasis. Although the mechanisms correlating neurodegeneration in PD and disturbed glucose homeostasis in the brain are still not well understood.

### 2.2. Implications of Mitochondrial Dysfunction and Disturbances in Cellular Bioenergetics of Glucose in PD

Oxidative phosphorylation occurs during cellular respiration [31]. It is involved in ATP or energy production and comprises two processes: electron transport chain (ETC) and chemiosmosis. Complex 1 is one of the four membrane-embedded protein complexes involved in electron chain transport (ECT), which consists of oxidation/reduction reactions to transfer electrons from one complex to another. The ETC builds a proton gradient in the inner mitochondrial membrane, which is then used to produce stored energy in the form of ATP. However, oxidative phosphorylation has the propensity to generate reactive oxygen and nitrogen species as a by-product, although this is relatively low under physiological conditions [32]. Decreased neuronal ATP levels have been observed in PD [15], which may need to be carefully interpreted since ATP measurements are a critical manoeuvre.

**Figure 1:**
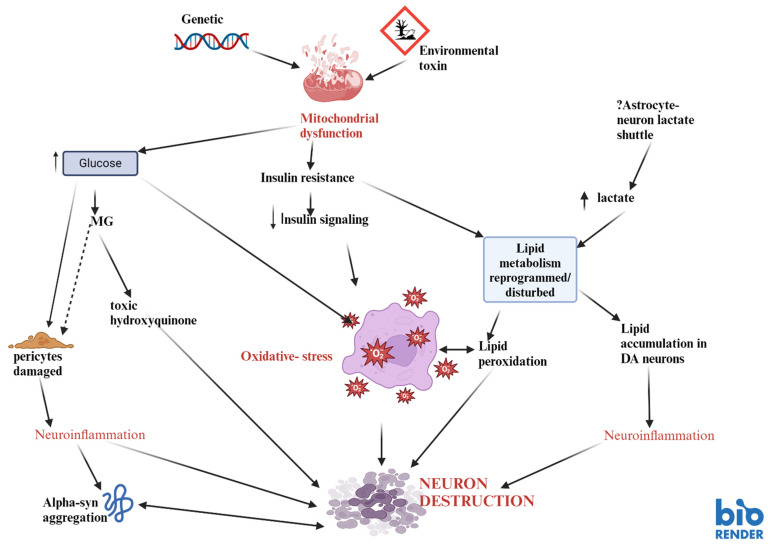
Metabolic dysfunction of glucolipid pathways in PD. Figure created using Biorender (https://www.biorender.com/). The metabolic dysfunction of glucose/lipid pathways may be of marked significance in the pathogenesis of the disease. The nigral faulty mitochondria in PD may be related to a genetic disposition or some environmental neurotoxin. This may disturb glucose metabolism and uptake, resulting in hyperglycaemia. Elevated glucose levels ↑ in the brain over a chronic period can have deleterious events. It can damage the pericytes in the blood-brain, resulting in a “leaky” blood-brain barrier, leaving the brain vulnerable to toxins and peripheral inflammatory mediators. The resulting neuroinflammation may favour the misfolding and accumulation of alpha-synuclein. Hyperglycaemia may contribute to the raised levels of its toxic metabolic by-product, methylglyoxal (MG), which can also damage the blood-brain barrier. This toxic metabolite may react with dopamine to produce toxic tetrahydro-isoquinoline, mediating apoptotic cell destruction. Due to a deficient mitochondrial complex 1 activity and disturbed glucose handling, may produce brain insulin resistance and disturbed insulin signaling. This would advocate cell destruction via free radical-mediated oxidative stress. Brain insulin resistance with decreased insulin signalling may disrupt lipid metabolism, leading to cytotoxic processes such as lipid peroxidation and oxidative stress. Additionally, the elevated brain lactate levels ↑ (perhaps due to a faulty astrocyte-neuron lactate shuttle) may also disturb or reprogram lipid breakdown. ↓ This may be responsible for the fat accumulation in the dopamine neurons, which may enhance their susceptibility to neuroinflammation. ? questionable pathway [35].

Mitochondrial dysfunction and deficiency in complex 1 can disturb energy-generating reactions such as oxidative phosphorylation, reducing cellular ATP levels and impeding cellular physiological functioning. In addition to that, it can also promote the production of free radicals that are toxic to cells [33]. Eventually, if these persist, cellular deleterious effects can be the result (Figure 1). In the presence of the inability of the cellular antioxidant defence system (such as glutathione peroxidase, glutathione, catalase, and superoxide dismutase) to overcome free radical production, resulting in oxidative stress, loss of cell membrane integrity, cell death, and cell senescence are the result. Therefore, the reduction in complex 1 activity [9] in the SN in PD represents a significant pathological factor. Hence, it may be associated with the dopaminergic neuronal defect characteristic of the disorder. The SN may be particularly vulnerable to decreased or malfunctioning mitochondria in light of its high mitochondrial density in order to provide a higher oxidative phosphorylation to meet the great ATP demands of the dopaminergic neurons [34].

### 2.3. Mitochondrial Genes and PD

There may also be an underlying genetic involvement in the neuronal energy deficit seen in the disorder. Mutation of the PD gene, *PGK1*, which encodes for phosphoglycerate kinase 1, a crucial enzyme required for glycolysis, increases the susceptibility to PD [36]. Conversely, augmentation of phosphoglycerate kinase 1 (PGK-1) activity alleviates PD symptoms [37] probably by providing ATP to release extracellular dopamine in the striatum [38]. PGK1 is a rate-limiting enzyme for the production of ATP in glycolysis in nerve terminals. More importantly, it has neuroprotective abilities since it permits the synapses to operate under conditions of hypoglycemia/hypometabolism [39]. This notion is supported by the modest repression of cell death of a PGK 1 activator, Terazosin, in many animal models [40]. This drug enhances PGK 1 activity for energy generation. It is regulated by a chaperone protein DJ-1 (PARK 7) or deglycase, and the PARK 7 gene mutations are associated with familial PD. Therefore, the neuroprotective role DJ-1′s might be twofold: it enhances PGK1 activity, thereby reducing hypometabolic defective synaptic function and hindering the accumulation of 3-phosphoglycerol-lysine resides in protein [41].

Although, significant pathophysiological heterogeneity has been demonstrated [42] in patients with PD, subtyping the disorder has been challenging [43]. Nevertheless, a recent study [44] suggests that idiopathic PD can be subdivided into two subtypes based on the severity and regional distribution of mitochondrial complex 1 deficiency in neurons. Furthermore, these subtypes also illustrate distinguishable cellular and clinical features.

More importantly, recent studies have highlighted the impact of complex 1 deficiency in the SN on the progression of the illness [45]. This reduction in mitochondrial complex 1 activity in PD patients versus controls concords with previous findings in asymptomatic PD/incidental Lewy body disease [23].

### 2.4. Is PD a “New Diabetes Type”—The Missing Link Between Insulin and Dopamine?

The seminal histochemical studies of Falck and Hellman [46], Lakomy and Chodkowska [47], and Cegrell [48] demonstrated the presence of catecholamines in pancreatic cells. Malaisse et al. [49] showed that insulin secretion can be stimulated by adrenergic receptors and acetylcholine and inhibited by alpha-adrenergic receptors. As summarised in detail by De Iuliis et al. [50] the presence of dopamine in secretory granules of pancreatic ß-cells leads to the assumption that hyperglycaemic effects might be driven by dopamine through insulin storage or the releasing mechanisms [50,51]. Indeed, tyrosine hydroxylase and aromatic amino acid decarboxylase have been identified in ß-cells, and monoamine oxidase is co-localized with insulin in secretory granules of ß-cells and VMAT1 (vesicular monoamine transporter 1) and DAT (dopamine transporter) were found in pancreatic cells [52,53] As well, D1- and D2-like dopamine receptors are expressed in ß-cells [54,55]. Thus, it seems that the role of dopamine in pancreatic insulin secretion is inhibitory and modulated by dopamine receptors [50].

Interestingly, there are no reports on the influence of levodopa treatment on pancreatic cells. It is unknown whether levodopa treatment of PD poses a risk for inducing/stimulating Diabetes mellitus or whether it is protective. Clinical and epidemiological studies are urgently needed to answer such and other related questions.

In the brain, insulin receptors have been identified in neurons and astrocytes of most brain regions, including the hippocampus, cerebral cortex, ventral tegmental area (VTA), and substantia nigra [50,56,57]. Insulin regulates glucose transporter (Glut) 4 and GLUT 8 [58]. Insulin triggers two pathways via insulin receptor substrate (IRS) docking proteins: the phosphoinositide-3-kinase cascade (PIK 3-involved in neuronal survival) and the Ras-Raf-mitogen-activated protein kinase (MAPK/ERK) signalling (involved in cell death) [50,59]. Furthermore, insulin promotes neuronal survival, plasticity and neuronal antioxidant defence as well as protection against neuronal apoptosis [60] via the SHC/ERK1/2 pathway [50].

**Figure 2:**
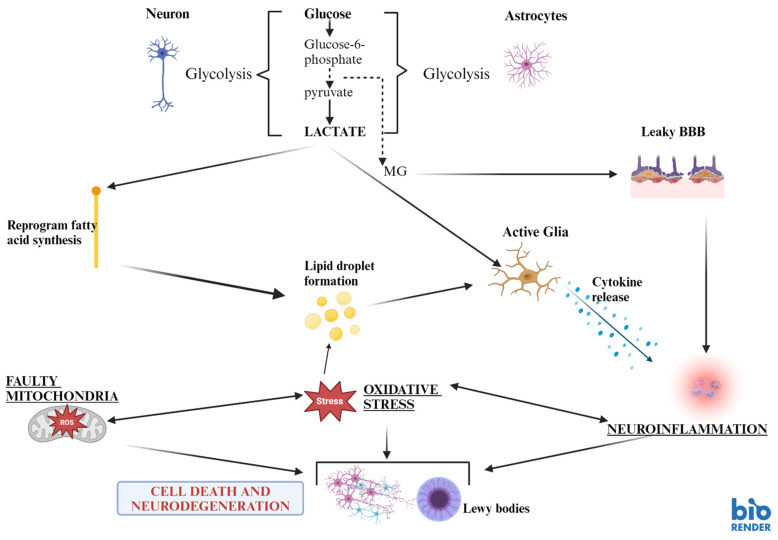
Lactate-related neurodegenerative pathways. Figure created using Biorender (https://www.biorender.com/). Lactate is produced from glycolysis of glucose. Elevated lactate levels in cerebrospinal fluid in parkinsonian patients may be related to the dysfunction of the astrocyte-neuron lactate shuttle or other factors related to the pathology of the disease. Lactate can trigger a number of pathways, prompting neuronal destruction. It can reprogram fatty acid synthesis, resulting in the production of lipid droplets, which can, in turn, activate glia and trigger neuroinflammation. Reactive oxygen species-mediated oxidative stress can also generate lipid droplets. Additionally, lactate can directly activate glial cells, causing the release of pro-inflammatory cytokines that can initiate neuroinflammation. Neuroinflammation, oxidative stress, and mitochondrial dysfunction can cause cellular destruction and accumulation of alpha-synuclein aggregates or Lewy bodies. Methylglyoxal (MG) is a toxic by-product of glycolysis; it can destroy the integrity of the blood-brain barrier, rendering it “leaky” and leaving the brain open to access to neurotoxins and inflammatory mediators that can induce neuroinflammation. Solid lines: major metabolic route; dashed lines: minor metabolic route [66].

These pathways have been shown to play an important role in both Alzheimer’s disease (AD) as well as in attention-deficit-hyperactivity-disorder (ADHD) [61]. Indeed, there is ample evidence pointing at the influence of glucose and insulin in mammalian Target of Rapamycin Complex 1 (mTOR C1) and Complex 2 (mTOR C2) pathways [61]. This might explain some of the processes happening in PD, too. Increased GABAergic inhibitory neurons in the striatum with upregulation of D1-receptor activity and downregulation of D2-receptors in medium spiny neurons is seen in transgenic mice, whose mTORC1 signalling is hyperactivated in inhibitory neurons in the striatum, while cortical neurons are left unaffected. These findings suggest critical involvement of the mTOR pathway in locomotor abnormalities in PD [62], (provisionally accepted work). <murine models of dopamine neuron-specific deletions of Raptor or Rictor, emphasize on the importance of both mTORC1 and mTORC2 for dopamine. Studies show that the inhibition of mTORC1 impacts dopamine neuron structure and function. This causes somatodendritic and axonal hypotrophy, increased excitability, and decreased dopamine production and release, which contrasts the consequences of inhibition of mTORC2 with some subtle effects on the output of VTA dopamine neurons [63]. The disruption of these pathways leads to pronounced deficits in dopamine release. Thus, this demonstrates their importance for dopaminergic functions [63]. In addition to that, rapamycin, an mTOR inhibitor and inducer of autophagy, potentiates oxidative stress-induced cell death, while the autophagy inhibitor 3-methyladenine protects the dopamine cells [64]. Dopamine receptors D2 and D3 are positive regulators of autophagy, while dopamine receptors D1 and D5 are negative regulators [65] (Figure 1 and Figure 2).

Of note in this regard is that a number of known drugs influence the mTOR pathways. For example, morphine that reduces the insulin receptor substrate/Protein kinase B (IRS/Akt) activity decreases K+ channel expression in the VTA, morphine increases mTORC1 and decreases mTORC2 signalling in the VTA [67,68]. In addition to that, striatal mTORC1 blockade may prevent D2-receptor-dependent extrapyramidal motor side effects of haloperidol in psychiatric illness [69,70].

In summary, both insulin and dopamine actions meet at the mTOR protein interaction, thus providing evidence for a robust linking of energy-providing pathways and neuronal dopaminergic activity in PD.

### 2.5. The Role of Hyperglycaemia in PD

The abnormal function of the mitochondria reported in PD is most likely directly related to the disturbed glucose metabolism and cellular bioenergetics. This notion is supported by the increased gluconeogenesis and abnormal changes in glucose breakdown in vivo seen in idiopathic PD patients [12,71,72]. Similarly, in Diabetes Mellitus, the pathological elevation of hepatic glucose formation accounts for the characteristic hyperglycaemia manifested in the condition [73]. In vivo and in vitro studies demonstrate the cytotoxic effects of uncontrolled hyperglycaemic levels. Indeed, long-term brain hyperglycaemia has been shown to inflict neurodegeneration, particularly to the nigrostriatal motor tract and mesocortical–limbic pathways in rats [74]. Culture studies using dopaminergic (pheochromocytoma) PC12 cells showed that the hyperglycaemic-related mode of action of dopamine neural destruction is exerted by oxidative stress by glycated modification [75,76] and apoptosis [77]. Furthermore, the elevated glucose levels in the diseased state account for the increased production of its toxic metabolite by-product, methylglyoxal. This is a 2-6-fold increase compared to physiological levels [35,78,79]. Alternatively, triacylglycerol hydrolysis can be generated from the lipid metabolic pathway [80].

Nevertheless, methylglyoxal intervenes in endothelial functioning and is instrumental in insulin resistance (IR) [81]. Under normoglycemic conditions, methylglyoxal is detoxified to lactate [82] by the actions of the two enzymes glyoxalases (1 and 2) in the presence of GSH (Figure 1). Therefore, the depleted GSH levels in the SN in PD [83,84] may exacerbate the toxic effects of methylglyoxal. It can exert cellular deleterious effects and inhibit cell growth via glycated modification by modifying proteins and lipids [85], disturbing calcium homeostasis and leading to cell destruction [86]. More importantly, methylglyoxal can augment the striatal dopamine depletion by reacting with dopamine to produce a toxic tetrahydro isoquinolin that mediates cellular destruction via apoptosis [79] and alpha-synuclein pathology-related glutaminergic hyperactivity [87].

Evidence from a meta-analysis [88,89] and epidemiological studies [90,91,92,93,94,95] showed that Type 2 diabetes increases the risk and development of idiopathic PD (by 1.21-fold). Indeed, PD patients with pre-existing Type 2 Diabetes suffer from a more rapid disease progression along with reduced survival rates. Interestingly, this seems unrelated to alpha-synuclein pathology [96]. Therefore, alpha-synuclein-containing LBs may be considered as a consequence of neuronal death rather than a cause.

Lixisenatide, a glucagon-like peptide agonist used in treating Diabetes Mellitus Type 2 and obesity, has been shown to have neuroprotective attributes in the PD mouse model [97]. Administration of lixisenatide to patients in the early stages of the course of PD (less than three years since diagnosis, responsive to treatment, and no motor complications) over 12 months modulated the progression of motor dysfunction [97,98]. The implications of these findings are fascinating and highlight the possible involvement of metabolic deficits or dysfunction in the pathogenesis and/or the progression of the illness. Although further studies are warranted with larger patient groups, different drug doses of lixisenatide are needed to access optimal efficacy coupled with minimum toxicity and longer duration.

### 2.6. The Effect of Cerebral Insulin Resistance in PD

Since Type 2 Diabetes is a consequence of peripheral insulin resistance (IR), IR in the brain might be observed in PD, pointing to IR as the common denominator for the two conditions [99,100,101]. Disruption in insulin metabolism or signalling in the brain may have enormous cellular consequences, given its vital role in brain function and neuronal survival [102]. These neuroprotective actions of insulin (Figure 1) are probably related to its ability to hinder the actions of cytotoxic processes such as oxidative stress, beta-amyloid toxicity, and apoptotic cellular destruction [103,104].

Perhaps peripheral IR and brain IR [105] might prompt brain pathologies, including neuroinflammation, oxidative stress [106] and a “leaky” blood–brain barrier (Figure 1) [107]. However, these cytotoxic processes are intertwined. Thus, pro-inflammatory cytokines released from a state of chronic neuroinflammation may also contribute to the central IR. Additionally, neuroinflammation may prompt or, at least partly, contribute to the alpha-synuclein pathology observed in the SN in PD. Interestingly, brain IR may contribute to alpha-synuclein pathology by blocking the insulin-degrading enzyme inhibition of alpha-synuclein fibril production, which subsequently supports its aggregation [108,109] and amyloid polypeptide [78]. Therefore, disturbed brain insulin signalling and IR can potentially induce neuronal destruction. The progressive loss of SN neurons in PD may, in part, subscribe to the disturbed IR/reduction in insulin production/glucose metabolism since the SN expresses insulin receptors [110,111] and the mid-brain contains detectable levels of insulin [112]. Indeed, reduced insulin receptors in the SN and greater IR have been found in PD [113]. The depleted brain insulin levels may hinder the release of dopamine from the striatum since insulin is involved in the action potential-dependent release of dopamine in this area [114].

Although central (alpha-synuclein aggregation and dopamine cell death) and peripheral (islet amyloid polypeptide aggregation and apoptosis) IR is associated with different pathological features, a so-called “cross-talk” or interaction between the two aggregates has been reported [115,116]. Amyloid polypeptide aggregation might support the abnormal accumulation of alpha-synuclein.

IR has been found to play a notable role in symptoms associated with metabolic- syndrome, such as elevation of lipid peroxidation and abnormalities in lipid profile [117]. Lipids play an essential role in cellular physiological and pathological processes.

### 2.7. Lipid Metabolism in PD

Lipid peroxidation occurs due to reactive free radical species-mediated oxidative stress. It oxidizes the lipids in cell membranes, breaking up the double bindings of the fatty acids that form the bi-lipid cell membrane, leading to the loss of cell membrane function and fluidity, resulting in cell death [118]. It is a marker for systemic IR and many neurodegenerative disorders such as PD [119] and Alzheimer’s disease [120,121]. Elevated lipid peroxidation indicates disturbed lipid metabolism, which may play an essential role in the pathogenesis and development of many disorders.

The SN in PD showed a reduction of polyunsaturated fatty acids, a substrate, and an index for lipid peroxidation [119]. Indeed, the elevation of lipid peroxidation was supported by the increase of an intermediate of this process, malondialdehyde. Thus, the decrease in polyunsaturated fatty acids is probably related to their utilization. The dysfunctional nigral mitochondria may be responsible for disturbing the lipid metabolism pathways in PD [122,123], since they modulate lipid metabolism and storage. On the other hand, lipid storage disorders also have the potential to disrupt mitochondrial function [14].

Lipid transport and regulation play a vital role in the physiological functioning of brain cells such as neurons and glia [124]. Interestingly, histological studies using a fluorescent probe (BODYIPY) displayed atypical lipid accumulation in dopaminergic neurons (Figure 1) and microglia (but not astrocytes) in the SN in PD [125]. The cellular preference for fat build-up is congruous with regions associated with neuroinflammation (Figure 1). This finding raises intriguing questions about the potential association of lipid accumulation and mitochondrial complex 1 reduction. Recently, statistically significant elevations in lipids such as cholesterol, sphingomyelin, and phosphatidylcholine have been described in the cerebrospinal fluid of PD patients [126]. The accumulation of lipids may pose a risk for disease pathogenesis or contribute to neurodegeneration via mechanisms such as free radical-mediated oxidative stress.

Free radicals might prompt the formation of lipids in neuronal cells. The lipids are transported by the glial–neuron lactate shuttle (via monocarboxylate transporters and fatty acid transport proteins) into the glia, where it is converted to lipid droplets [127]. The monocarboxylate transporter permits lactate secretion from the glia. Afterward, lactate is taken up by the neurons and converted to pyruvate and acetyl-coenzyme A. Subsequently, the breakdown product is used for the synthesis of fatty acids. Consecutively, they are transported via fatty acid protein to the glia. Moreover, lactate is not only a breakdown product of glycolysis but plays a crucial role in neurodegeneration [66] and induces metabolic reprogramming [128]. The latter is demonstrated by the elevated lactate concentration in the cerebrospinal fluid of PD patients compared to control subjects, which corresponds to the progression of the disease [129]. The neurodegenerative effects of lactate (Figure 2) might be mediated by the activation of glia, which in turn releases pro-inflammatory cytokines (such as Interleukin-1beta and 6, tumour necrosis factor-alpha), initiating the cytotoxic neuroinflammatory cascade [24]. Its production might be increased by the upregulation of hexokinase-2 in dopaminergic neurons in PD. This might promote degenerative processes such as apoptosis and alpha-synuclein aggregation [130]. This notion is supported by inhibiting hexokinase-2 expression, which decreases lactate production and related apoptotic destruction of dopamine neurons [130].

Lactate can prompt lipids to produce and mobilize lipid droplets (Figure 2) [128,131]. Lipid droplets execute essential physiological and pathological roles in energy homeostasis by mitigating harmful cellular effects [132]. Furthermore, oxidative stress in the brain promotes the accumulation of lipid droplets in glial cells [133,134,135]. Its ability to trigger inflammation might confer its pathological nature. Since defective lipid homeostasis, signalling, and lipid droplet accumulation have been associated with an increased risk of PD, metabolic disturbances might play a vital role in the development of the disease.

### 2.8. Bioenergetic Drug Treatment in Parkinson’s Disease

Therapies used for the treatment of Type 2 Diabetes may be promising candidates to supplement dopaminergic drug treatment strategies in PD.

As reported by De Iuliis et al. [50] the compound “Exenatide”, a Glucagon-like Peptide 1 (GLP-1) analogue, improved motor behaviour [41,111,136,137,138]. In addition to that, Athauda et al. [139,140] found higher IRS1 phosphorylation and Akt and mTOR expression in the individuals who received Exenatide, which was associated with better motor scores.

DPP-4 (Dipeptidyl Peptidase-4) is an enzyme that plays a role in glucose metabolism. DDP4 inhibitors block the enzyme dipeptidyl peptidase-4. Therefore, they could be used to treat Type 2 Diabetes since they increase insulin production and reduce the amount of glucose produced by the liver. This helps lower blood sugar levels in people with Type 2 Diabetes. Common DPP-4 inhibitors include medications like Sitagliptin, Saxagliptin, and Linagliptin. DPP-4 inhibitors lower the risk for the development of PD [141,142].

Thiazolidinediones (TZDs) improve insulin sensitivity in peripheral tissues such as muscle and fat. They activate PPAR-γ (peroxisome proliferator-activated receptor gamma), which regulates glucose and lipid metabolism. Thiazolidinediones significantly decrease the incidence of PD [143].

Pioglitazone is a type of TZD. There has been some interest in pioglitazone for its possible neuroprotective effects, potentially decreasing the risk or progression of PD. However, the evidence is not definitive, and more research is needed to confirm this potential. Pioglitazone showed no significant decline in the incidence of PD [141], but a reduction in the incidence of PD was reported when administered together with statins [144].

Ping et al. [145] describe that the commonly prescribed medication for Type 2 Diabetes, metformin, may be associated with an increased incidence of PD. Metformin influences mitochondrial function and glucose metabolism, which may play a role in neurodegenerative processes happening in PD. However, more research is required to fully understand the mechanisms and the risks.

The water-soluble vitamin thiamine (Vitamin B1) is essential for energy metabolism and the functioning of the nervous system. It is critically involved in glucose metabolism in the Krebs cycle and is required for the synthesis of neurotransmitters. Thiamine deficiency can lead to neurological conditions such as Wernicke–Korsakoff syndrome and Beriberi, and it has been suggested that thiamine could have a neuroprotective role in other conditions as well. In the context of PD, there is ongoing research into the potential protective role of thiamine, as it supports mitochondrial function and neuronal health. While thiamine deficiency has been linked to cognitive impairments, its supplementation has been investigated for its therapeutic effects in neurodegenerative diseases like PD, with promising results [30,146].

Thiamin is a cofactor of several enzymes active in the glycolysis pathway. Therefore, thiamine has been connected to the PD pathology and candidate pathways which involve transcription factors Sp1, p53, Bcl2, caspase-3, tyrosine hydroxylase, glycogen synthase kinase-3ß, vascular endothelial growth factor, advanced glycation end products, nuclear factor kappa B, mitogen-activated protein kinase, and the reduced form of nicotinamide adenine nucleotide phosphate [147,148]. Indeed, high-dose thiamine can effectively reverse PD motor and non-motor symptoms [30]. This is in line with experimental studies in the rat model of MPTP-induced Parkinsonian in rats [149] and experimental thiamine deficiency [124,150].

## 3. Discussion

Parkinson’s disease (PD) is a devastating neurological disorder with a number of subtypes (e.g., early-onset and late-onset PD, tremor-dominant, akinetic-rigid PD, PD with tremor, rigidity and bradyphrenia, PD with dementia, Lewy body dementia, vascular PD) [151,152]. The triggers underlying PD are unknown, although several have been described in recent years, e.g., carbon monoxide, insecticides, pesticides, chemicals, viruses, iron, manganese, plumbum, and genetic mutations [153,154]. Oxidative stress and inflammation, as well as mitochondriopathy, have been discussed as being of major pathological importance [20,62,155]. Therefore, we have focused on comorbid disorders that might be triggering the molecular cascade leading to PD. We have focused on metabolic disease and Type 2 Diabetes to investigate them as possible risk factors prompting, e.g., early-onset PD.

The ultimate goal of this review is to unravel molecular aspects of PD and to open up new avenues for treatment in order to minimize these risk factors. Type 2 Diabetes (DM) comprises several aspects of interest as risk factors for triggering PD. (1) This is of interest because disturbances in the glucose and lipid metabolism seen in DM lead to the loss of metabolites important for the brain’s energy supply. Indeed, a loss of ATP production in mitochondria has been suggested to be responsible for mitochondriopathy in PD, leading to the degeneration of nigrostriatal dopaminergic neurons. (2) The role of Type 2 Diabetes has been extensively studied in Alzheimer’s Disease (AD) ([60,122,156] for review) and conclusions from such research can be of importance for PD, too. (3) Thiamine (Vitamin B1) is a key player in glucose metabolism and is vitally involved in mitochondrial membrane development and synaptic membrane function [28]. Thiamine might show therapeutic benefits in mitochondrial respiratory chain deficit [146]. Thus, it is of significance to further elucidate the link between glucose, insulin, lipids, and dopamine with respect to cellular bioenergetic (hyperglycaemia, insulin resistance) dysfunction in mitochondria. Bioenergetic drug treatment strategies are of interest to enlarge the armamentarium to fight PD. Here, we report in detail on all these mechanisms Therefore, we extend previous work on this topic in PD. In addition to that, the review uncovers evidence from the molecular investigation, which may lay the foundation for clinical studies designed to improve the brain’s energy metabolism in patients with PD and in patients with DM and/or disturbances of lipid metabolism. Potential promising clinical applications [157] should be investigated further with a focus on glucose metabolism to open up new and exciting possibilities for innovative therapeutic interventions in PD. Hence, this report should stimulate further efforts to elucidate biochemical aspects of PD other than, e.g., alpha-synuclein-related pathology. Given the increasing evidence reported, the latter might even be a result of cellular energy loss, oxidative stress, inflammation, and mitochondriopathy.

In summary, this overview emphasized on the important role of energy loss due to deficits in the glucose/lipid metabolism in the pathogenesis of PD. This is critical for understanding the molecular mechanisms discussed in PD, like oxidative stress, inflammation, mitochondriopathy, and the pathological processes and interactions induced by alpha-synuclein. The review summarizes molecular evidence and wants to open up new clinical strategies to fight the molecular underpinnings that lead to the devastating disorder PD.

## 4. Conclusions

The mitochondrial dysfunction described in PD may be closely linked to irregular glucose metabolism and brain IR. Injecting recombinant complex 1 or NADH-quinone oxidoreductase adeno-associated virus into the SN pars compacta of MPTP-treated mice has shown promising results. This treatment significantly improved dopamine neuronal survival and motor disabilities potentially by compensating for the compromised complex 1 activity [22]. The implications of this are significant, suggesting that such a treatment could be effective in the prodromal phase of idiopathic PD, offering hope for salvaging the remaining neurons from the pathological processes.

Brain hyperglycaemia in PD might be closely associated with insulin depletion, malfunction in glucose metabolism, or perhaps the inability of cerebral insulin to cope with the pathological elevation of glucose. Consequently, vulnerable regions in the brain, such as the SN, are susceptible to the onslaught of cellular destruction processes. Elevated brain glucose may elevate the production of its cytotoxic metabolite methylglyoxal, which is related to dopamine depletion, IR, and apoptotic cellular destruction in susceptible mid-brain areas.

It seems that the alterations in the peripheral/central IR pathways have the propensity to facilitate neuronal destruction. This might be primarily related to the loss of the neuroprotective role of insulin in PD. This reflects the operation of complicated pathways that prompt many questions worth exploring further: Is PD a metabolic disorder? Does the peripheral IR lead to central IR or vice versa? How does IR lead to neuronal death? In PD, is the brain IR a consequence of faulty nigral mitochondrial complex 1? Is there a shared pathogenesis between PD and DM? What factors support or confer the development of PD in Type 2 Diabetic patients? Are there other neurodegenerative disorders that exhibit similar processes as in Type 2 Diabetes?

It is becoming increasingly clear that PD and Type 2 Diabetes share common pathophysiological processes. Metabolic disturbances are central to these mechanisms. Ultimately, other factors such as environmental/neurotoxins/genetic factors may finally determine the development of PD in some patients with Diabetes. This underscores the importance of understanding and addressing metabolic disturbances in these diseases.

The nigral mitochondrial dysfunction in PD may consequently disturb the regulation of lipid homeostasis metabolism and promote cellular degenerative processes. Altered lipid metabolism/storage might impact cellular bioenergetics and insulin signalling and disturb glucose/lipid pathways. Other factors may also be involved in disruption in the lipid homeostasis. Modified glucolipid pathways may contribute to PD’s elevated lactate levels in the cerebrospinal fluid. The increased lactate from neurons and glia may be considered an important therapeutic target to reduce catastrophic cellular destruction since it activates the neuroinflammatory pathways.

The impairment in glucose metabolism may disturb the functioning of the blood–brain barrier. Hyperglycaemia can disturb pericyte function and damage the tight junction features in the barrier via oxidative stress and neuroinflammation/inflammation [158,159]. Additionally, IR depletes the pericyte population [160]. Pericytes are an essential constituent of this physical barrier. They exert an integral role in the physiological functioning of the microglia, which provide integrity and vascular supply to the blood–brain barrier. The raised lipids in the cerebrospinal fluid from PD patients may also support a disrupted blood–brain barrier (Figure 2).

It appears that perhaps the focus needs to be shifted from mechanisms underlying abnormal alpha-synuclein aggregation in the brain toward the disruption of the glucolipid metabolic pathways to provide a better understanding of the complex pathogenesis of PD. The disease may have a multisystemic pathogenesis coupled with multifactorial factorial (genetic risk factors, environmental, neurotoxin exposure) interactions such asenergy deficits, particularly in the synapses. This might be a (predisposing) vulnerability factor for the degenerating dopamine neurons. PGK 1 may represent a critical pharmacological point of action for exercising bioenergetic control for neuroprotection and modulation. Additionally, antidiabetic drugs may prove to be a potential game changer in delaying the progression of PD.

Brain insulin resistance may disrupt lipid metabolism, leading to cytotoxic processes such as lipid peroxidation and oxidative stress. Additionally, the elevated brain lactate levels (perhaps due to a faulty astrocyte–neuron lactate shuttle) may also disturb or reprogram lipid breakdown. This may be responsible for the fat accumulation in the dopamine neurons, which may enhance their susceptibility to neuroinflammation.

Methylglyoxal is a toxic by-product of glycolysis; it can destroy the integrity of the blood–brain barrier, rendering it “leaky” and leaving the brain open to access to neurotoxins and inflammatory mediators that can induce neuroinflammation.

## Data Availability

The original contributions presented in the study are included in the article, further inquiries can be directed to the corresponding authors.
